# Editorial: The hierarchical organization of supramolecular systems: From fundamentals to biomedical applications, Volume II

**DOI:** 10.3389/fbioe.2023.1177799

**Published:** 2023-03-13

**Authors:** Chenxuan Wang, Lei Liu, Xiaoguang Wang

**Affiliations:** ^1^ State Key Laboratory of Medical Molecular Biology, Institute of Basic Medical Sciences Chinese Academy of Medical Sciences, School of Basic Medicine Peking Union Medical College, Beijing, China; ^2^ Institute for Advanced Materials, Jiangsu University, Zhenjiang, Jiangsu, China; ^3^ William G. Lowrie Department of Chemical and Biomolecular Engineering, The Ohio State University, Columbus, OH, United States; ^4^ Sustainability Institute, The Ohio State University, Columbus, OH, United States

**Keywords:** self-assembly, intermolecular interaction, biomaterials, nanotechnology, colloidal chemistry

Self-assembly is a new frontier of chemistry. By constraining the covalent and noncovalent interactions of molecular building blocks, self-assembly organizes molecules to form ordered structures on different length scales. Highly specific supramolecular architectures have been identified within a variety of biologically relevant systems, such as the complementary base pairing in DNA replication, the folding and assembly of polypeptides, the ligand-receptor recognition and signal transduction, and the micellization of lipids. Inspired by the supramolecular structures in nature, numerous functional supramolecular materials have been designed and fabricated in a bottom-up manner, leading to a wide range of technological applications. To date, this research field has intersected with physics, biology, and material science, becoming a multidisciplinary scientific community. The key scientific issues to be addressed in this research field include, but are not limited to, 1) the controllable multi-level, multi-component molecular self-assembly and assembly dynamic processes and 2) the additivity, cooperativity, and directionality of intermolecular interactions between molecules. Thanks to the distinct biochemical and physical characteristics of supramolecular materials, the bottom-up approach of molecular self-assembly has been widely used to engineer novel biomedical materials, offering opportunities for improving disease diagnosis, treatment, and prognosis.

This Research Topic was organized to include 5 Original Research articles and 2 Review articles that reflect recent efforts made toward the fundamentals and biomedical applications of the hierarchical organization of supramolecular systems. This Research Topic aims to attract the wide interest of the readers of the Frontiers series of journals to explore the forefront of supramolecular science and construct novel supramolecular materials for biomedical applications. The papers published in this Research Topic cover the regulation mechanisms governing polypeptide association, the hierarchical self-assembly of multi-component nanomaterials, and naturally occurring supramolecular systems and their potential medical applications.

Understanding the driving forces behind the evolution of the sophisticated biologically relevant hierarchical organization provides crucial insight into the principles that guide the design of supramolecular systems. Ma et al. focused on the peptide-based coacervation process, where multivalent interactions drive peptides to form a dense liquid phase from a dispersed solution. Coacervates can be formed by either the self-assemblies of peptides or the co-assemblies of peptides, nucleic acids, and polysaccharides. Coacervation is known to play a role in a variety of physiological and pathological processes, such as the formation of membranless organelles, transcriptional regulation, and proteopathy. Recent findings have shed light on the thermodynamic and kinetic mechanisms behind coacervation and have paved the way for applying coacervates in novel therapeutic approaches, including DNA/small molecule drug delivery platforms, mRNA-based vaccines, and biomimetic protocells. Zhang et al. investigated the intercellular transfer of RNA by extracellular vesicles (EVs), i.e., lipid-bound assembled structures that are naturally released from prokaryotes and eukaryotic cells that are as small as a hundred nanometers. They isolated the EVs derived from high glucose-induced monocytes and identified the microRNA (miR-142-5p) shuttled by the EVs as a key participant in diabetic endothelial dysfunction. This discovery motivates the administration of miR-142-5p inhibitors as a potential strategy for protecting the cardiovascular system from diabetic damage.

Motivated by the hierarchically assembled structures in nature, diverse supramolecular structures have been synthesized in the lab with striking biophysical and physiochemical properties inspiring the development of the next-generation of biomaterials. Qiu et al. synthesized Fe_3_O_4_-based magnetic nanoparticles (NPs) and functionalized NPs with polythiolester. The thiolester groups on the NP surfaces are active to covalently capture and enrich free amines from tissue samples. By employing these polythiolester-modified NPs to facilitate the chemoselective capture of amine derivatives, mass spectroscopy-based analysis methods have been upgraded to rapidly detect more than 100 amines in lung adenocarcinoma cell lines, mouse organ tissues, and 103 human serum samples with high-throughput and high-coverage. In addition, Xie et al. prepared a surface-enhanced Raman scattering (SERS) substrate by embedding gold nanoparticles (AuNPs) within the metal-organic frameworks, MIL-101(Cr). This substrate exhibited a promising potency as a sensor for detecting volatile organic compounds (VOCs) by SERS. The detection limit of this sensor was determined to be 6 ppm for toluene, 5 ppm for 4-ethylbenzaldehyde, and 75 ppm for formaldehyde, respectively. By mimicking the structures of a natural extracellular matrix, the aggregated nanofibers and networks formed by fibronectin and collagen can be engineered with tunable mechanical properties and used as substrates for a three-dimensional (3D) cell and organoid culture. Following these breakthroughs in 3D organoid culturing, Song et al. reviewed the clinical translation of patient-derived organoids in individualized therapy.

The interfaces between biological molecules and supramolecular materials are important as they play a foundational role in regulating the *in vivo* and *in vitro* activities of biomaterials. Wang Z. et al. functionalized the surfaces of fibrinolytic agent-loaded microbubbles with fibrin-targeted CREKA/rhPro-UK, achieving an enhanced sonothrombolysis in the endovascular low-frequency ultrasound treatment. Wang M. et al. used the affinity peptide for human epidermal growth factor receptor 2 (HER2) to modify magnetic NPs and developed a semiquantitative fluorescent method for HER2 immunostaining on circulating tumor cells (CTCs) by using the peptide-functionalized magnetic NPs. This method shows merit in the diagnosis and prognosis of first-line advanced breast cancer, where the value of the CTC-HER2 status determined by the method can help predict the efficacy of anti-HER2 treatment.

Overall, this Research Topic reviews the recent progress made in the biomedical applications of supramolecular materials. We hope this Research Topic can encourage communication between different disciplines to promote the development of supramolecular science and technology. It should be noted, however, that although a variety and abundance of assembled systems have been designed and fabricated in a lab, the level of structural complexity is still simple relative to the hierarchical, multifunctional structures existing in nature. In the future, efforts is needed to unravel the mechanisms behind the self-organization of biomolecular architectures to facilitate the *de novo* design of supramolecular materials.

